# Salinomycin induced ROS results in abortive autophagy and leads to regulated necrosis in glioblastoma

**DOI:** 10.18632/oncotarget.8905

**Published:** 2016-04-21

**Authors:** Enric Xipell, Marisol Gonzalez-Huarriz, Juan Jose Martinez de Irujo, Antonia García-Garzón, Fred F. Lang, Hong Jiang, Juan Fueyo, Candelaria Gomez-Manzano, Marta M. Alonso

**Affiliations:** ^1^ The Health Research Institute of Navarra (IDISNA), Pamplona, Spain; ^2^ Program in Solid Tumors and Biomarkers, Foundation for the Applied Medical Research, Pamplona, Spain; ^3^ Department of Pediatrics, University Hospital of Navarra, Pamplona, Spain; ^4^ Department of Biochemistry University of Navarra, Pamplona, Spain; ^5^ Brain Tumor Center, UT MD Anderson Cancer Center, Houston, TX, USA

**Keywords:** glioblastoma, autophagy, regulated necrosis, ROS production

## Abstract

Glioblastoma is the most frequent malignant brain tumor. Even with aggressive treatment, prognosis for patients is poor. One characteristic of glioblastoma cells is its intrinsic resistance to apoptosis. Therefore, drugs that induce alternative cell deaths could be interesting to evaluate as alternative therapeutic candidates for glioblastoma. Salinomycin (SLM) was identified through a chemical screening as a promising anticancer drug, but its mechanism of cell death remains unclear. In the present work we set out to elucidate how SLM causes cell death in glioblastoma cell lines (both established cell lines and brain tumor stem cell lines), aiming to find a potential antitumor candidate. In addition, we sought to determine the mechanism of action of SLM so that this mechanism can be can be exploited in the fight against cancer. Our data showed that SLM induces a potent endoplasmic reticulum (ER) stress followed by the trigger of the unfolded protein response (UPR) and an aberrant autophagic flux that culminated in necrosis due to mitochondria and lysosomal alterations. Of importance, the aberrant autophagic flux was orchestrated by the production of Reactive Oxygen Species (ROS). Alleviation of ROS production restored the autophagic flux. Altogether our data suggest that in our system the oxidative stress blocks the autophagic flux through lipid oxidation. Importantly, oxidative stress could be instructing the type of cell death in SLM-treated cells, suggesting that cell death modality is a dynamic concept which depends on the cellular stresses and the cellular mechanism activated.

## INTRODUCTION

Of all primary brain tumors, glioblastoma is the most aggressive. Despite the rigorous treatment, recurrence occurs in most patients, and median survival is just 14.6 months [1–3]. One feature that characterizes glioblastoma is that its cells show an intrinsic resistance to apoptosis [4, 5]. Therefore, drugs that induce cell death in ways other than apoptosis are alternative therapeutic candidates for glioblastoma, and the study and evaluation of such drugs will potentially provide useful knowledge.

The three main cell death modalities described for cancer cells are apoptosis, autophagic cell death and necrosis [6]. In the context of tumor development, apoptosis, which is associated with specific morphological cell changes [7], can be understood as acting as a barrier to tumor growth [8]. Macroautophagy (herein autophagy) is a cellular resistance mechanism with the ability to recycle cellular organic material with the objective to produce energy, when autophagy is overloaded, it could culminate with cell death, a phenomenon referred to as *autophagy-associated cell death* or *autophagy-mediated cell death* [9, 10]. Cell death by necrosis can occur in several ways, all of which lack the features of apoptosis or autophagy-associated cell death. This modality includes a broad variety of molecular pathways with specific morphologic features: cytoplasmic swelling, rupture of the plasma membrane, swelling of organelles, and moderate chromatin condensation [11]. Cell death by necrosis can be “programmed” - in which case it is referred to as *regulated necrosis* - or not. Despite the considerable amount of information that has been obtained on the subject of regulated necrosis cell death, no definitive markers have been identified, and, therefore, the main distinguishing criteria for necrosis cell death are the lack of both apoptosis and autophagy-associated cell death [10].

Salinomycin (SLM) is a coccidiostat that has proven to be a highly effective agent at killing not only bulk tumor cells but also cells in the recalcitrant cancer stem cell compartment [12]. Despite the well-known antitumor effect of SLM, the mechanism by which SLM brings about cell death remains poorly understood. Several reports have addressed the question of the modality of cell death induced by SLM, but there is still no consensus: some authors have proposed apoptosis, others autophagic cell death and others necrosis [13–16].

SLM acts as an ionophore for K^+^ and Na^+^ ions [17], which means that the cellular concentrations of these cations will be balanced by SLM, thereby altering membrane potentials (ΔΨ), such as the mitochondrial membrane potential (ΔΨ_m_), and that of the lysosome through these ions movement [18]. It is rational to think that SLM brings about cell death by inducing mitochondria and lysosome dysfunction due to the loss of membrane potentials, which in both organelles involves Na^+^ and/or K^+^ [19, 20]. The above considerations, we believe, make SLM a particularly interesting candidate drug to evaluate in glioblastoma.

In the work we report here, we set out to elucidate how SLM causes cell death in glioblastoma cell lines. Understanding the biological underpinnings of SLM-induced cell death could aid in designing more effective and less toxic therapeutic strategies, whether based on SLM itself or not, for glioblastoma. In our experimental system, SLM was at the cross roads of various different modalities of cell death, and study of SLM shed much light on the various mechanisms and processes involved.

## RESULTS

### SLM induces a potent antitumor effect in brain tumor stem cells (BTSCs) and established adult and pediatric glioma cell lines *in vitro*

**Figure 1:**
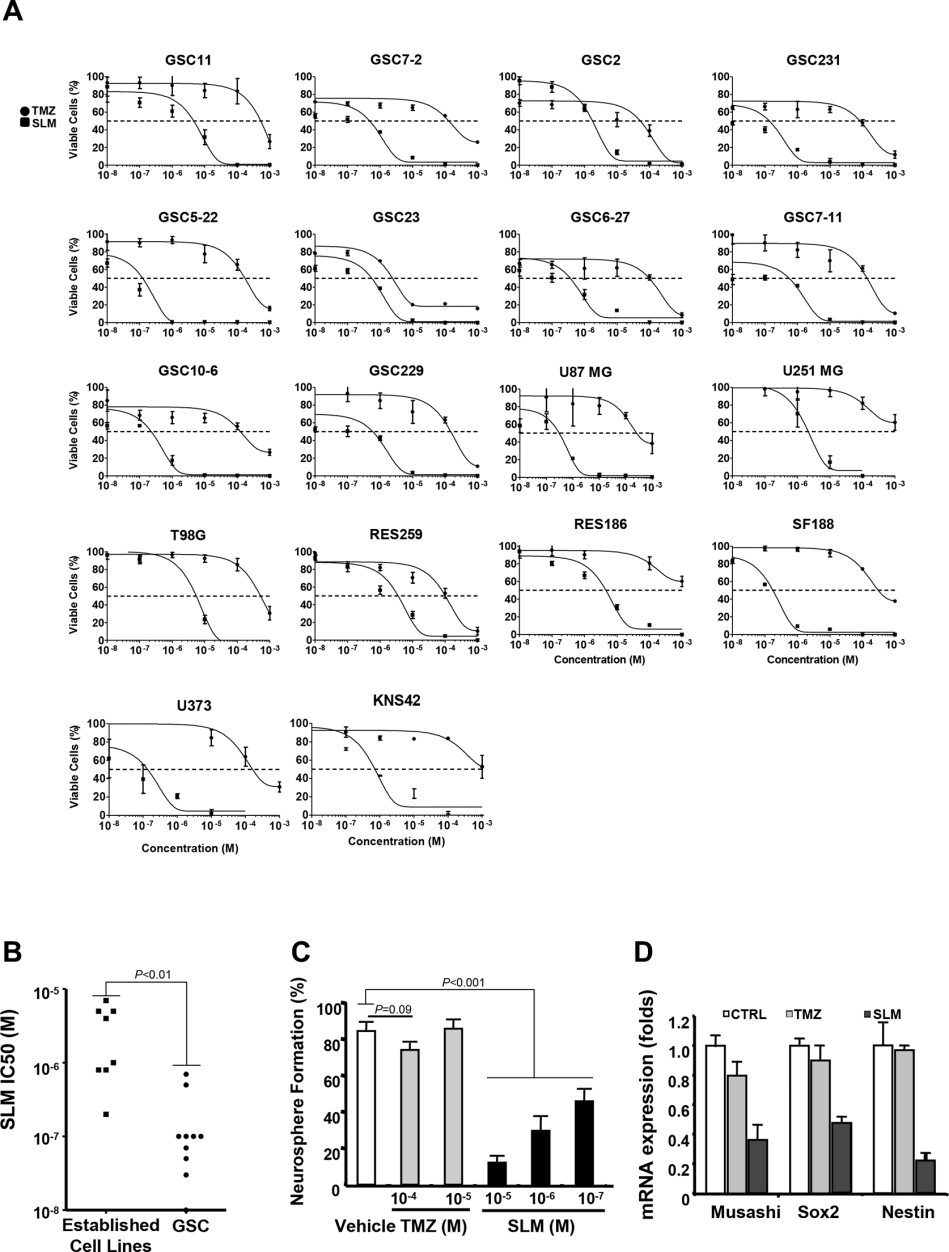
SLM exerts a potent anti-glioma effect *in vitro* and reduces GSC self-renewal capacity (**A**) Cells were seeded at a density of 5·10^3^ cells per well in 96-well plates. The following day, cells were incubated with either TMZ or SLM at a concentration ranging from 10^−3^ M to 10^−8^ M. Seven days after treatment, cell viability was assessed using MTT assays. The results are expressed as mean values ± SD from three independent experiments and are represented as cell viability relative to non-treated cells (whose viability was taken to be 100%). (**B**) Median-effect doses (IC50s) of SLM in attached cell lines and neurosphere culture. An IC50 is the median-effect dose (the dose causing 50% of cells to be affected, which is equivalent to 50% survival). The results are expressed as mean values from Figure 1A. (**C**) GSC11 BTSCs were treated with TMZ or SLM at the indicated concentrations. The number of secondary spheres generated was assessed after 10 days and expressed as relative to non-treated cells (= 100%). To confirm that the spheroids were formed by stem cells, we randomly selected at least 15 individual secondary spheres and subjected them to further, long-term (two-months), propagation in each subcloning experiment. (**D**) Expression of different stem-cell markers by Q-RT-PCR in GSC11 cells treated with TMZ or SLM. RNA was extracted 72 hrs after treatment and Q-PCR analysis was performed. *GAPDH* was used as an internal control. To determine relative gene expression, we used the comparative threshold cycle method.

First, we assessed the cytotoxic effect of SLM *in vitro* in several glioma stem cell (GSC) lines and in established adult and pediatric glioma cell lines comparing it with that of temozolomide (TMZ), the first-line treatment for glioma. SLM had a lower half-maximal inhibitory concentration (IC_50_) than TMZ in all the cell lines tested regardless of differentiation status (Figures 1A and S1A and Table 1).

**Table 1:** Median-effect doses (IC50) of salinomycin or temozolomide in glioma cell lines

IC50		
	SLM	TMZ
**GSC11**	5·10^−7^ M	8·10^−4^ M
**GSC23**	1·10^−7^ M	2·10^−6^ M
**GSC7–2**	5·10^−8^ M	2·10^−4^ M
**GSC6–27**	1·10^−7^ M	3·10^−6^ M
**GSC5–22**	1·10^−8^ M	2·10^−4^ M
**GSC2**	1·10^−7^ M	1·10^−5^ M
**GSC231**	6·10^−8^ M	1·10^−5^ M
**GSC7–11**	1·10^−7^ M	3·10^−4^ M
**GSC10–6**	3·10^−8^ M	2·10^−4^ M
**GSC11–28**	7·10^−8^ M	4·10^−4^ M
**GSC229**	7·10^−7^ M	3·10^−4^ M
**U87 MG**	5·10^−6^ M	6·10^−4^ M
**T98G**	7·10^−6^ M	6·10^−4^ M
**U373**	8·10^−7^ M	4·10^−4^ M
**U251 MG**	4·10^−6^ M	out of range
**PBT7**	2·10^−7^ M	6·10^−4^ M
**KNS42**	8·10^−7^ M	out of range
**RES186**	5·10^−6^ M	out of range
**RES259**	1·10^−6^ M	1·10^−4^ M
**SF188**	2·10^−7^ M	6·10^−4^ M

In view of the suggestion that SLM preferentially targets the cancer stem cell compartment [12], we compared SLM IC_50_ in GSCs versus established cell lines. SLM had lower IC_50_ in GSCs than established cultures (Figure 1B). Moreover, the self-renewal capacity, which is an intrinsic property of stem cells, was markedly reduced in SLM-treated BTSCs relative to TMZ-treated BTSCs; similarly, neurosphere size was smaller and markers of undifferentiation were drastically reduced in SLM treated BTSCs when compared with TMZ (Figures 1C–1D and S1B–S1D).

In summary, *in vitro* SLM exerts a robust cytotoxic effect in established glioma cell lines and BTSC cell lines.

### SLM treatment does not trigger apoptosis and blocks the autophagy flux in glioma cell lines

**Figure 2:**
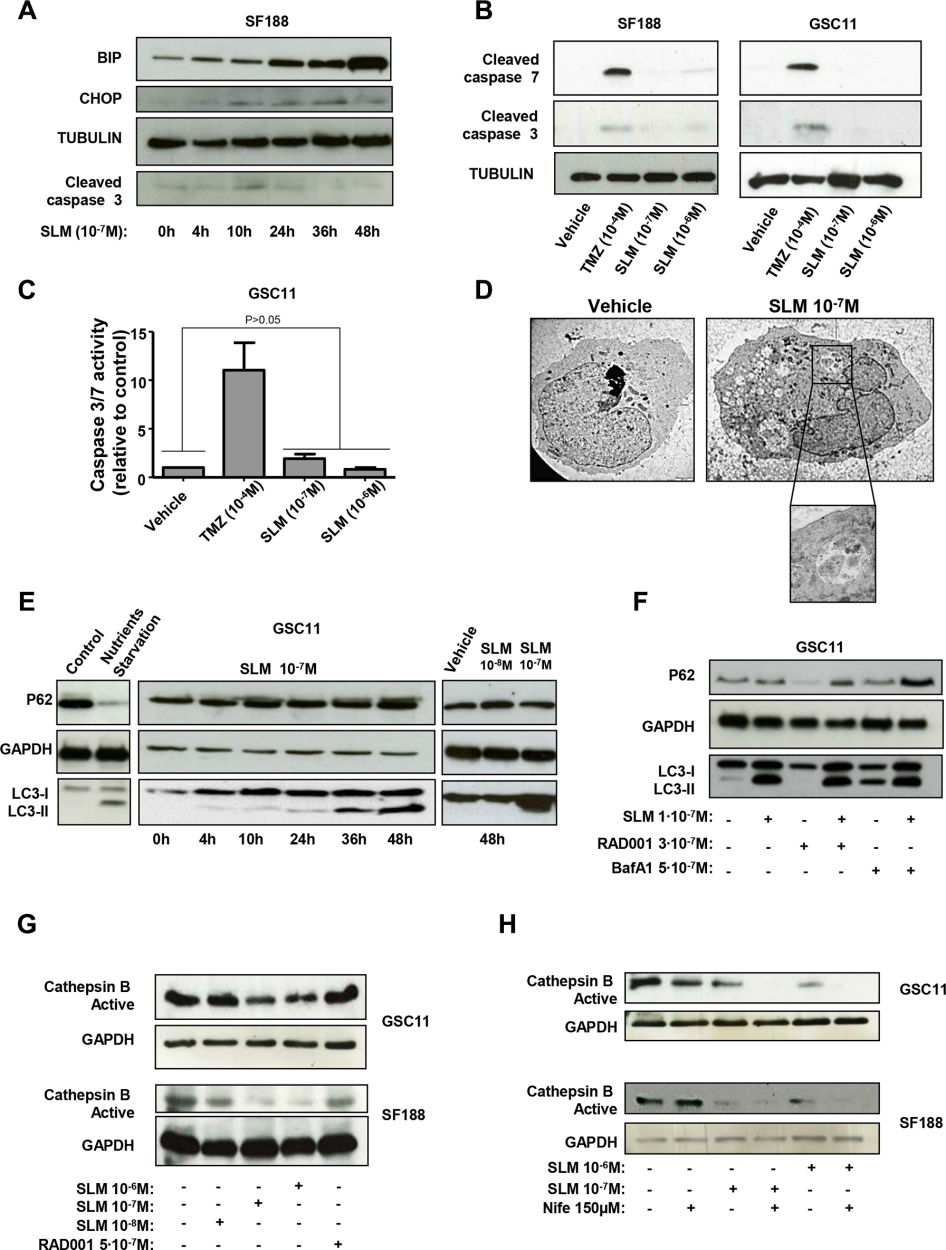
SLM induces ER stress and an aberrant autophagic flux (**A**) SF188 cells were seeded at a density of 1 × 10–5 cells per well in a 6-well plate. The following day, cells were incubated with SLM (1 × 10^−7^ M) and collected at the indicated times. Samples were analyzed by western blot for BiP, CHOP and cleaved caspase 3. α-Tubulin was used as loading control. The western blot shown is representative of three independent experiments. (**B**) SF188 and GSC11 cells were incubated with SLM (1 × 10^−8^ M or 1 × 10^−7^ M) or TMZ (1 × 10^−4^ M). Cells were collected after 48 h, and samples were analyzed by western blotting for cleaved caspase 7 and cleaved caspase 3. Tubulin was used as the loading control. The western blot shown is representative of three independent experiments. (**C**) GSC11 cells were seeded at a density of 5 × 10^−3^ cells per well in 96-well plates. The following day, cells were incubated with either TMZ or SLM at the indicated concentration. 48 hours after treatment, caspase −3 and −7 activities were measured with the Caspase-Glo^®^ 3/7 Assay. The results are expressed as mean values ± SD from three independent experiments and caspase −3 and −7 activities in treated cells are represented relative to the corresponding activities in non-treated cells. (**D**) Transmission electron microscopy analysis. GSC11 cells were treated with SLM (1 × 10^−7^ M) and harvested 48 h later. The micrographs shown are representative of the morphologic features observed (1600× magnification). (**E**) GSC11 cells were incubated with SLM (1 × 10^−8^ M or 1 × 10^−7^ M) and collected after different incubation times. Samples were analyzed by western blotting for p62 and LC3-I to LC3-II conversion. GAPDH was used as the loading control. The western blot shown is representative of three independent experiments. Nutrient-starved cells were used as a positive control for autophagy. (**F**) GSC11 cells were seeded at a density of 1 × 10^5^ cells per well in 6-well plates. After 24 h of culture, cells were incubated with RAD001, SLM or bafilomycin A1 (BafA1) at the indicated concentrations. Cells were collected 48 h later and subjected to western blot analyses. The western blot shown is representative of three independent experiments. (**G**) GSC11 and SF188 cells were incubated with SLM and RAD001 at the indicated doses. Cells were collected 48 hours after treatment, and samples were analyzed by western blotting for active cathepsin B; GAPDH was used as the loading control. (**H**) GSC11 and SF188 cells were incubated with Nife (1, 5 × 10^−4^ M) alone or in combination with SLM (10^−7^ M or 10^−8^ M). Cells were collected after 48 h, and samples were analyzed by western blot for active cathepsin (B) GAPDH was used as the loading control. The western blot shown is representative of three independent experiments.

It has been shown that SLM treatment results in the upregulation and activation of several key Endoplasmic Reticulum (ER) stress proteins [21], including the multifunctional proteins BIP and CHOP [22]. In our study, we observed an increase in BIP and CHOP levels for 48 h after treatment with SLM, indicating ER stress and activation of the Unfolded Protein Response (UPR) (Figure 2A). CHOP is a transcription factor which promotes apoptosis [23, 24]; however, we did not detect activation of effector caspases in cells treated with SLM (Figures 2A–2C and S2A). We further confirmed the absence of apoptosis evaluating FITC-Annexin V and PI staining in SF188 and GSC11 cell lines after treatment with different SLM concentrations. The results showed that SLM treatment did not increase the number of apoptotic cells when compared with untreated cells. Administration of TMZ significantly increased the percentage of apoptotic cells in comparison with control or SLM-treated cells (Figure S2B). These data allowed us to rule out apoptosis as the mechanism of cell death induced by SLM.

Next we interrogated whether SLM induces autophagic cell death. Treatment of glioma cells (GSC11 and SF188) with SLM resulted in a moderate but significant increase in the percentage of acidic vesicles when compared with vehicle or TMZ treated cells, (18 ± 6.3% and 21 ± 4% for SLM 10^−7^ and 10^−6^M, respectively for GSC11 cell lines and 21 ± 3.5% and 21 ± 7% for SLM 10^−7^ and 10^−6^M, respectively for SF188) (Figure S2C). We proceeded to assess cellular morphology by electron microscopy. TEM images showed that there were more intracellular vesicles in SLM-treated cells than in untreated cells and confirmed the formation of autophagosomes, autolysosomes and/or lysosomes in cells treated with SLM (Figure 2D). At the biochemical level, we observed time and dose dependent LC3-I to LC3-II conversion in SLM treated cells; this LC3 lipidation suggests an increase in autophagosome synthesis (Figures 2E and S2D). The amount of p62 protein, however, was not reduced by SLM treatment (Figures 2E and S2D). These data suggest insufficient degradation of the cargo in autolysosomes and, therefore, a block in the autophagic flux.

To gain a deeper understanding of the process, we combined SLM with either bafilomycin A1 (BafA1; a specific vacuolar H^+^-ATPase inhibitor), which is an inhibitor of autophagy, or RAD001 (everolimus), which promotes autophagy. Treatment with either SLM alone or BafA1 alone led to an accumulation of LC3-II and p62. Combined treatment with SLM plus BafA1 resulted in an even greater accumulation of p62, suggesting that some degree of p62 degradation occurred in cells treated with SLM alone. As expected, treatment with RAD001 alone resulted in a decrease in the amount of p62 levels. Combined treatment with SLM and RAD001 resulted in normal amounts of p62, indicating that SLM blocked the p62 degradation driven by RAD001 (Figures 2F and S2E).

SLM can act as an ionophore for Na^+^ and K^+^ therefore it could lead to an unstable Donnan potential and therefore interfere with lysosome maturation (Figure S3A). The maturation of cathepsin B requires an acidic environment that is only obtained in mature lysosomes [25]. For this reason, active cathepsin B is a possible marker for lysosome maturation. We observed that treatment with SLM led to a decreased in active cathepsin B levels in a concentration and time dependent manner (Figures 2G and S3B). Active cathepsin B decrease was not observed in cells treated with the positive autophagy stimuli RAD001.

Next, we treated cells with a combination of SLM and Nifedipine (Nife), which is a voltage sensitive calcium-channel blocker [26]. Cells treated with a combination of SLM plus Nife had dramatically fewer amount of acidic vesicles than untreated cells and cells treated with SLM alone (Figure S3C). Cells treated with SLM plus Nife had less active cathepsin B than cells treated with SLM alone (Figure 2H), which suggests that SLM plus Nife blocked acidification and in turn the maturation of cathepsin B.

Altogether the above results indicate that combination of both drugs blocks the acidification and in turn the maturation of cathepsin B. In addition, the impaired autophagic flux observed in SLM-treated glioma cells can be attributed to diminished lysosome activity

### SLM induces necrosis cell death

We proceeded to determine whether the cell death induced by SLM could be through necrosis. We examined the most widely-accepted executioners of necrosis: low levels of intracellular ATP (energetic catastrophe), lysosome membrane permeability (LMP) and osmotic swelling [27, 28].

**Figure 3:**
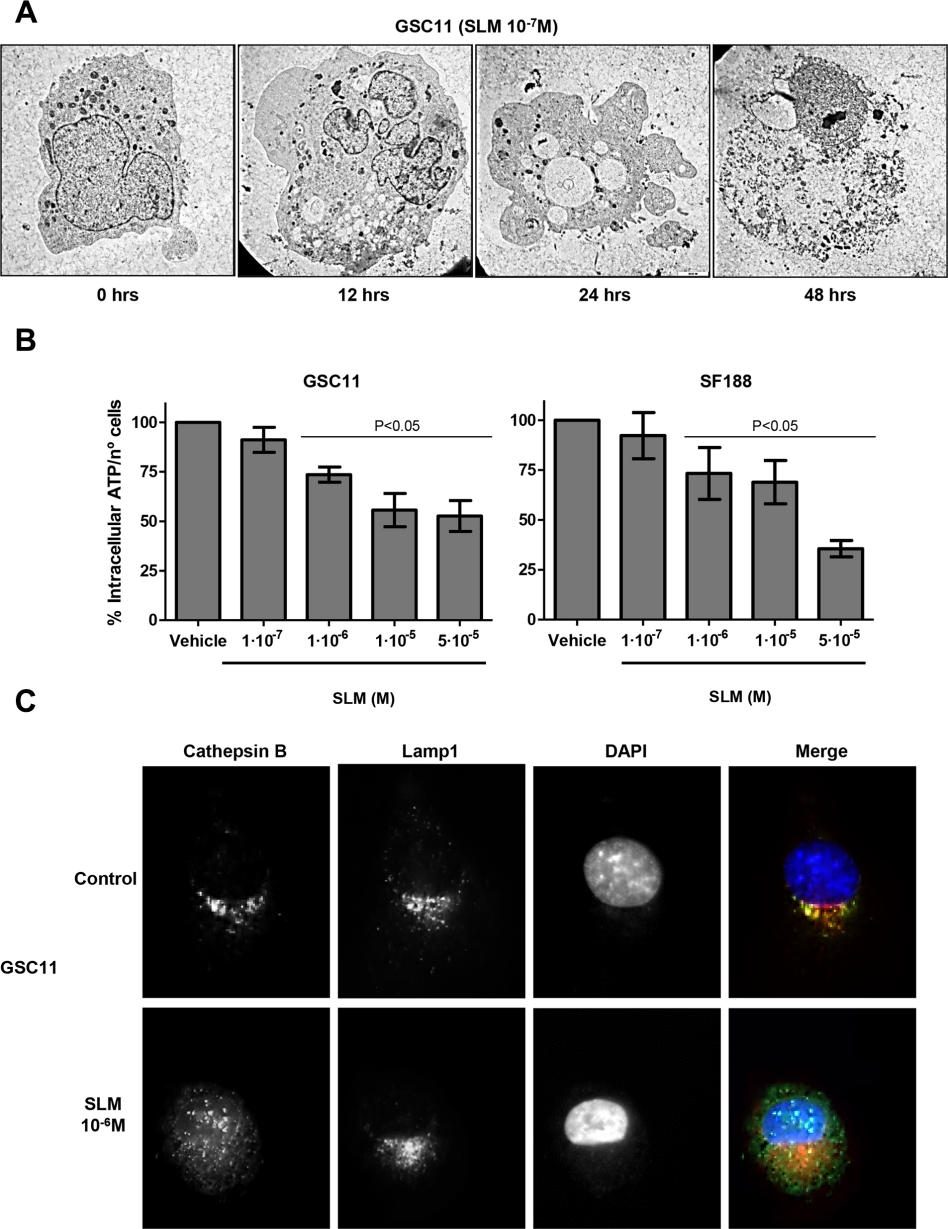
SLM induces necrosis (**A**) GSC11 cells were treated with SLM (10^−7^ M), and cells were collected 0, 12, 24 and 48 hours later. The micrographs shown are representative of the morphology of treated cells (× 1000 magnification). (**B**) GSC11 and SF188 cells were seeded at a density of 1 × 10^5^ cells per well in a 6-well plate. The following day cells were incubated with SLM and 48 hours later the levels of ATP were measured. Data are presented as the mean ± SD of three independent experiments. (**C**) GSC11 cells were incubated with SLM, at the indicated concentrations, and, 48 hours later, cathepsin B (green) and Lamp1 (red) localization were assessed with immunofluorescence. Representative images for three independent experiments are shown; DAPI (blue) was used for nuclear staining.

A time-dependent analysis of cell morphology after SLM treatment corroborated an increment of autophagosomes in cells in the early stages of treatment (*T* = 24 h). At later times SLM-treated cells displayed moderate chromatin condensation and cytoplasmic swelling with rupture of the plasma membrane, both of which are features associated with necrosis (Figure 3A) [11, 28]. The observed cell morphology confirmed that apoptosis was not the mechanism of cell death. In addition, cells treated with increasing doses of SLM displayed a decrease in intracellular ATP levels (Figure 3B). We studied cathepsin B localization, which indicates the degree of LMP [29], and observed that treatment with high doses of SLM (5 × 10^−6^ M) resulted in the release of cathepsin B from lysosomes (Figures 3C and S4A). Altogether, these data suggest that SLM induced regulated necrosis in glioblastoma cell lines.

### SLM alters ΔΨ_m_

**Figure 4:**
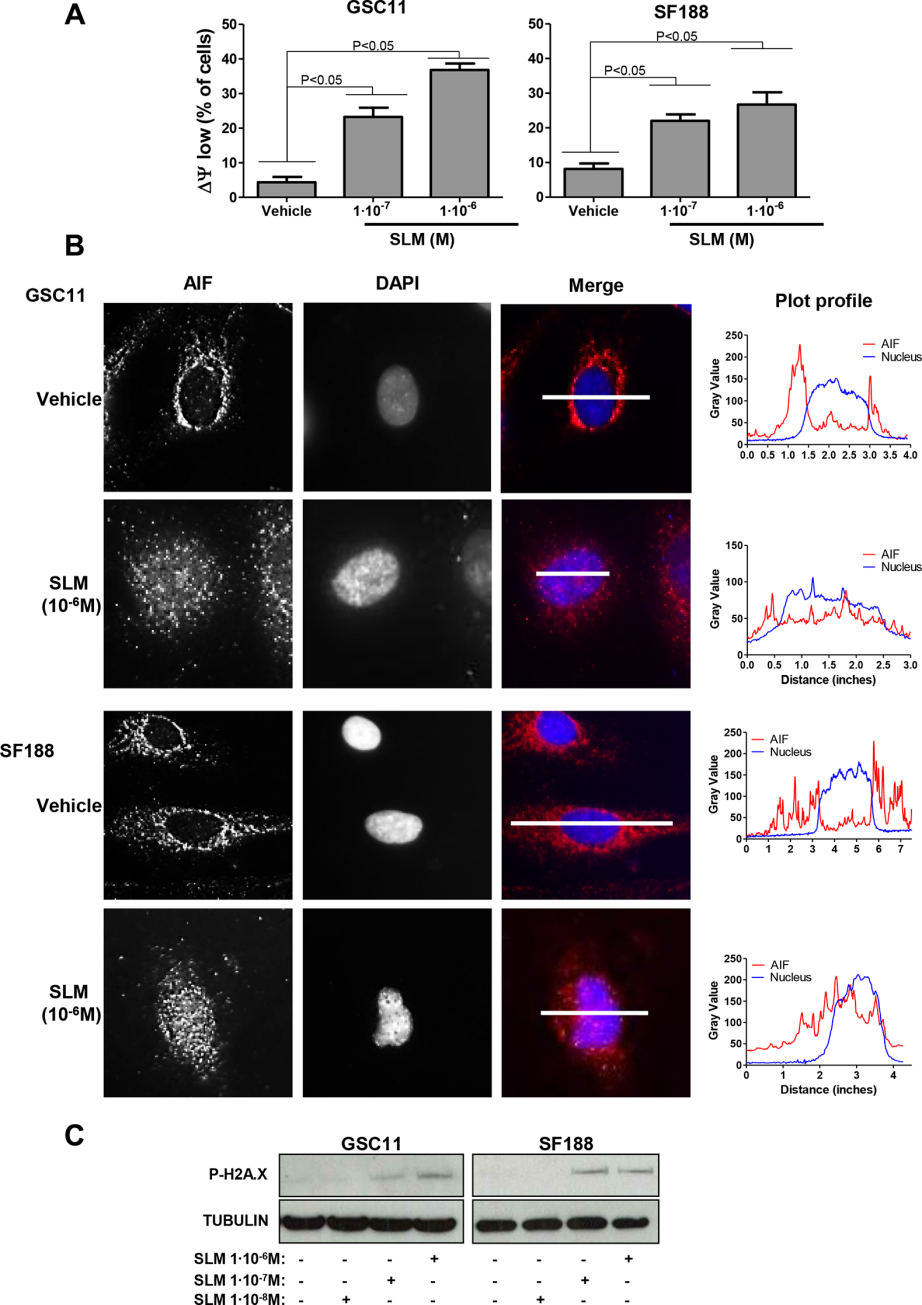
SLM induces mitochondrial MOMP (**A**) GSC11 and SF188 cells were seeded at a density of 1 × 10^5^ cells per well in 6-well plates and were incubated with SLM at the indicated concentrations. Cells were collected 48 hours later, stained with Rhodamine 123 (10 μM) and analyzed by flow cytometry. The percentage of cells with low ΔΨ_m_ was determined. Data are given as the mean ± SD of three independent experiments. (**B**) GSC11 and SF188 cells were treated with SLM at the indicated concentrations, and 48 hours later samples were collected and fixed. AIF (red) localization was evaluated by immunofluorescence. Representative images of three independent experiments are shown. DAPI (blue) was used for nuclear staining. The micrographs shown are representative of the morphology of treated cells (×400 magnification). The right panel is a plot of the profile along the grey line drawn in the merged micrographs, where the x-axis is distance along the line and the y-axis is pixel intensity. (**C**) GSC11 and SF188 cells were seeded at a density of 1 × 10^5^ cells per well in a 6-well plate. Cells were incubated the next day with SLM (10^−7^ M). Samples were collected three days later, and protein expression levels were analyzed by western blotting using antibodies against P-H2A.X. The loading control was α-tubulin. The western blot shown is representative of three independent experiments.

Because SLM has been shown to alter ΔΨ_m_ [30], we turned our attention to mitochondria. SLM treated cells displayed significantly lower ΔΨ_m_ than untreated cells (*P <* 0.05; Figure 4A), indicating the possibility of alteration in mitochondrial functionality. Next, we evaluated the mitochondrial outer membrane permeabilization (MOMP), which could trigger cell death monitoring the localization of the mitochondrial protein AIF [31]. Immunofluorescence analyses revealed that high doses of SLM resulted in release of AIF from mitochondria into the cytoplasm from where it spread and entered the nucleus (Figure 4B). AIF is an endonuclease and, if in the nucleus, could induce DNA damage [32]. We observed double strand breaks in SLM-treated cells (Figure 4C) and it is possible that this DNA damage is induced as a consequence of AIF internalization.

In summary, our data reveals that SLM can disrupt the ΔΨ_m_ and induce MOMP with the consequent release and spreading of AIF.

### SLM induced ROS modulates the UPR and the autophagy flux in glioma cells

**Figure 5:**
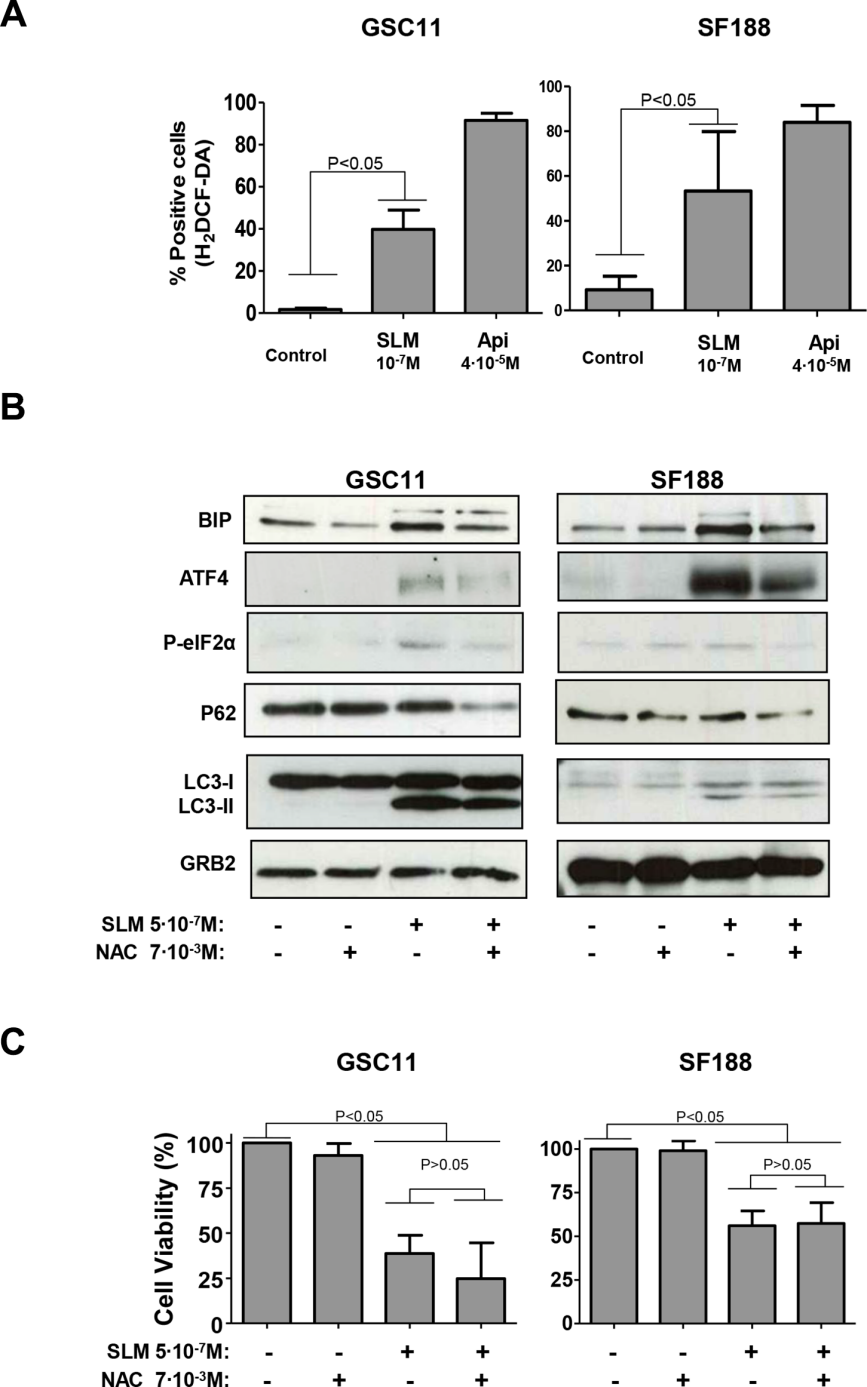
SLM treatment induced oxidative stress (**A**) GSC11 and SF188 cells were seeded at a density of 1 × 10^5^ cells per well in 6-well plates. Next day cells were incubated with SLM or Apigenine (Api; as a positive control) at the indicated concentrations. Cells were collected 48 hours later and stained with H2DCF-DA (4 μM). Cells were then analyzed by flow cytometry. (**B**) GSC11 and SF188 cells were seeded at a density of 1 × 10^5^ cells per well in a 6-well plate. The following day, cells were incubated with SLM, NAC or both drugs at indicated doses. Cells were harvested three days later, and protein samples were analyzed by western blot for BIP, ATF4, P-elF2α, p62 and LC3-II conversion. GRB2 was used as loading control. Shown is a representative western blot of three independent experiments. (**C**) GSC11 and SF188 cells were seeded at a density of 1 × 10^5^ cells per well in a 6-well plate. The following day cells were incubated with SLM, NAC or both drugs at the indicated doses. Three days later cells were counted in a Neubauer chamber. The results are represented as the percentage of cell viability relative to non-treated cells; the mean ± SD from three independent experiments is shown.

Another important consequence of disruption of the ΔΨ_m_ is an increment in oxidative stress [33]. We found SLM to trigger, in a concentration dependent manner, an increased in the amount of ROS (Reactive Oxygen Species) in glioma cell lines (Figure 5A). Apigenin (Api) was used as a positive control since it is a well-known H2DCF-DA oxidative agent [34].

To better understand the role of ROS in our model, we used N-Acetyl-cystein (NAC) to reduce the oxidative stress brought about by SLM [35]. Cells treated with SLM plus NAC had lower levels of the protein ER stress markers BIP, P-PERK and P-elF2α than cells treated with SLM alone. These results indicate that there is a decrease in the UPR when we reduced the amount of ROS with NAC (Figures 5B). Regarding autophagy, LC3-II levels remained constant in cells treated with SLM, regardless of whether the cells were also treated with NAC, and this indicates that the formation of autophagosomes occurred independently of the induced oxidative stress. Importantly, p62 levels were lower in cells treated with SLM plus NAC when compared with cells treated with SLM alone (Figures 5B and S5A). Finally, the addition of NAC to the SLM treatment did not reduce the extent of cell death (Figures 5C and S5B). These results suggest that SLM induced an autophagic process but also increased the production of ROS, which could have been interfering with the autophagic flux; addition of NAC, however, seemed to allow autophagic flux to continue and finally induce cell death.

### ROS modulates autophagy flux in SLM-treated cells through lysosome membrane permeability (LMP)

**Figure 6:**
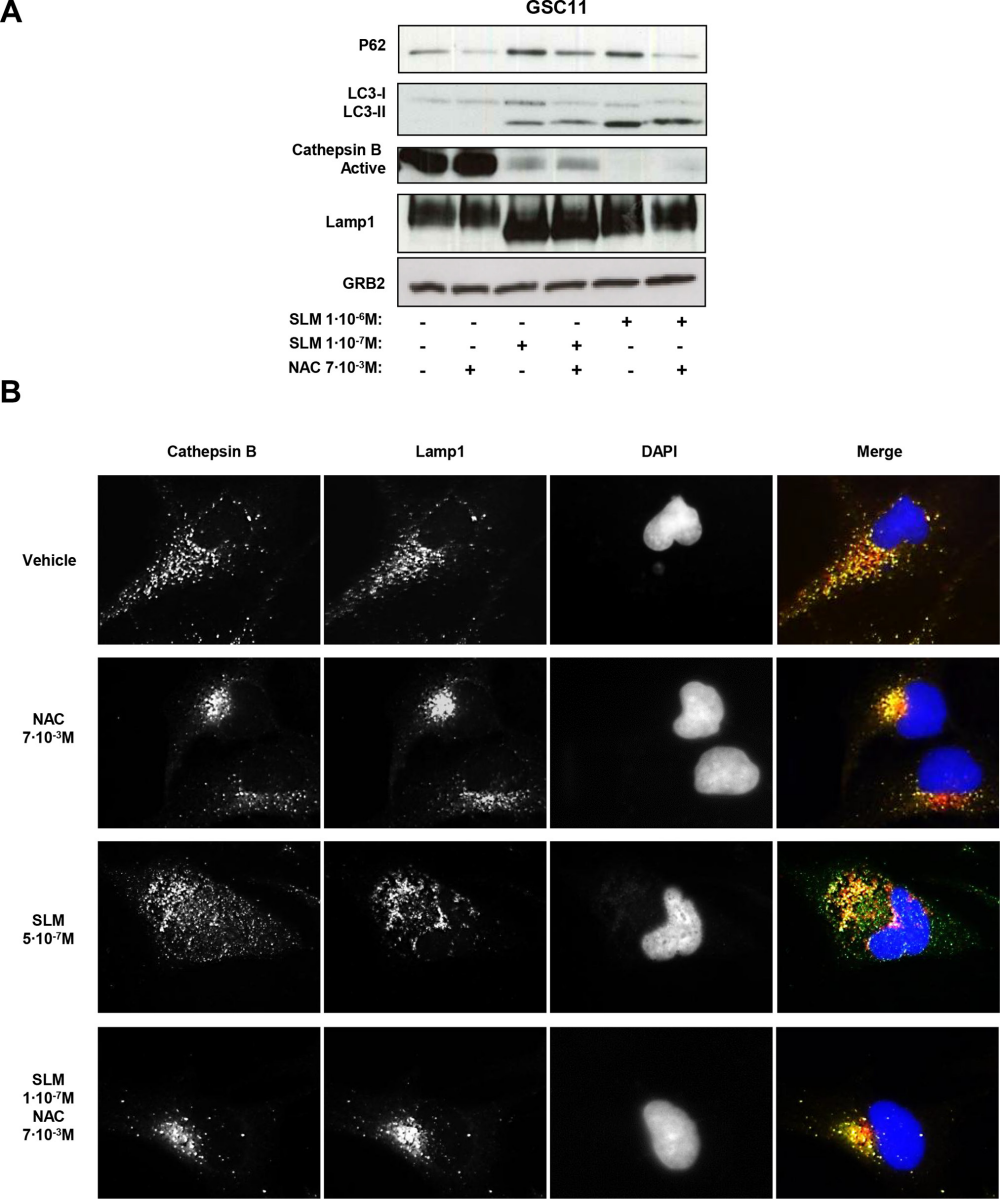
Oxidative stress modulates autophagic flux and lysosomal permeabilization (**A**) GSC11 cells were seeded at a density of 1 × 10^5^ cells per well in a 6-well plate. Cells were incubated the next day with SLM and/or NAC at the indicated doses; samples were collected 48 hours later, and protein expression levels were analyzed by western blot using antibodies against p62, LC3-II conversion, active cathepsin B, and Lamp1. α-tubulin was used as loading control. The western blot shown is a representative of three independent experiments. (**B**) GSC11 cells were incubated with SLM, NAC, or both at the indicated concentrations. After 48 hours of drug administration, cathepsin B (green) and Lamp1 (red) localization were assessed with immunofluorescence. Representative fluorescent images for three independent experiments are shown; DAPI (blue) was used for nuclear staining.

In order to validate our data, we analyzed the levels of active cathepsin B after treatment with SLM/NAC. Interestingly, the levels of active cathepsin B decreased after SLM treatment even when NAC was also present. The amount of p62, however, was lower with NAC (Figure 6A). The implication is that even though NAC addition did not restore active cathepsin B levels, there was sufficient residual lysosomal activity to degrade p62.

**Figure 7:**
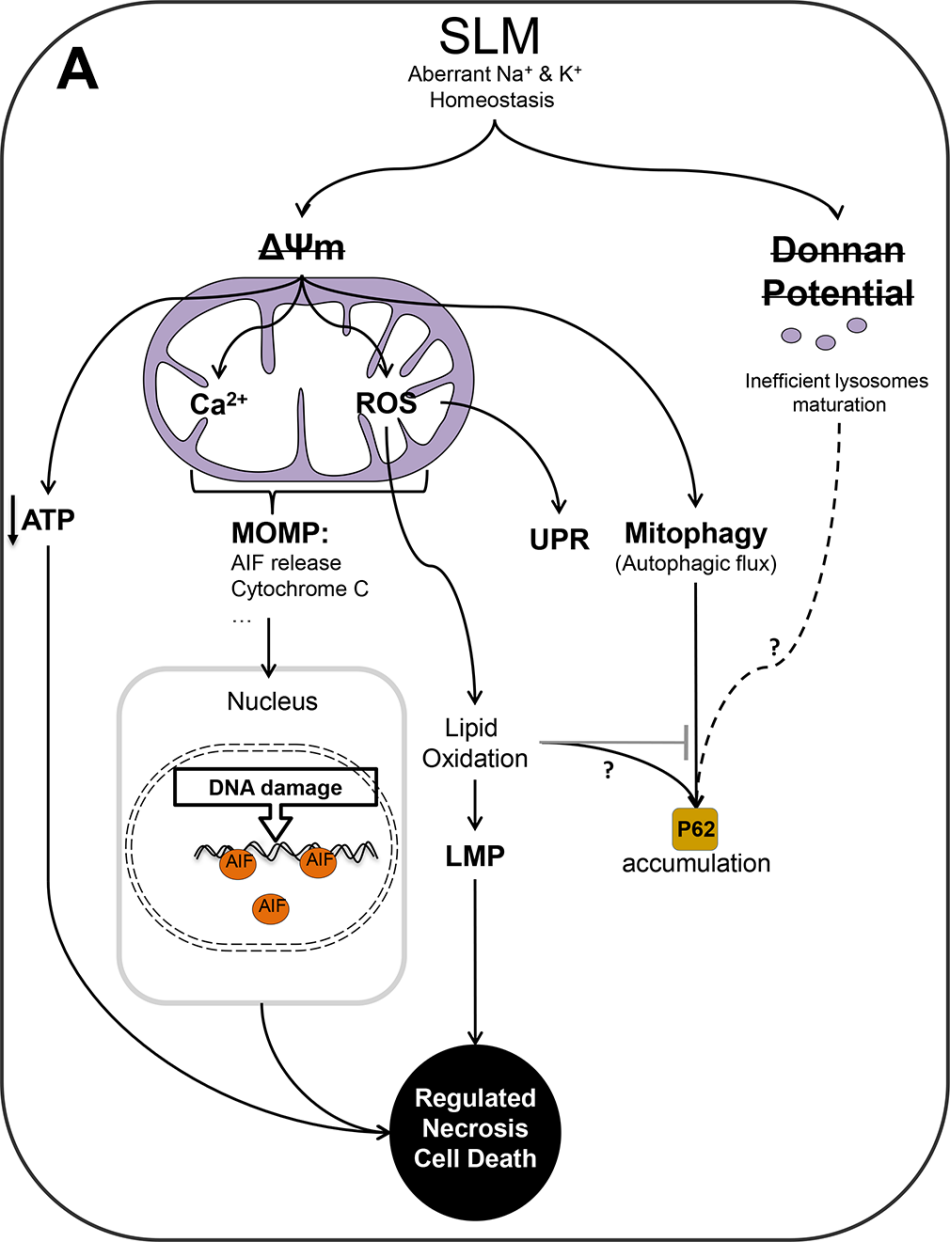
Schematic representation of the authors' proposed model of the action of SLM in glioblastoma cells (**A**) SLM induces a direct alteration in mitochondrial ΔΨ and as consequence a disruption in the Donnan potential of the lysosomal membrane. SLM thus partially inhibits lysosome maturation leading to a reduction in the amount of active cathepsin (**B**) Although there is no doubt that SLM induces an aberrant autophagic flux, the role of the Donnan potential in this respect is not clear. In the case of mitochondria, SLM induced a decrease in the ΔΨ and an increase in the levels of intra-mitochondrial calcium and ROS. In our proposed model, this oxidative stress triggers the UPR. The elevated levels of calcium and ROS inside mitochondria results in MOMP which leads to release of AIF. Once AIF spreads into the nucleus, it damages DNA through its endonuclease activity. The presence of AIF would establish a feedback loop that would cause cell death by a mechanism of regulated necrosis. In parallel to this, we propose that it should not be ruled out that the action of SLM involves other different molecular routes that could lead to regulated necrosis. For example, a mitochondrial membrane alteration would directly cause a deficit of ATP, which would cause energetic failure, which would, in turn, provoke cell death by regulated necrosis. In addition, impairment of mitochondrial activity would trigger mitophagy, with engulfment of the organelle and initiation of autophagy. A key role in our proposed model is played by oxidative stress, which blocks autophagic flux, probably through a process involving lipid oxidation.

Finally, we evaluated LMP as a possible phenomenon that could be interfering with the autophagic flux. In physiological conditions cathepsin B is localized inside of the lysosomes therefore we assessed the localization of cathepsin B. We used lamp 1 as lysosomal marker. Control (untreated) cells showed a co-localization of cathepsin B and lamp 1. SLM-treated cells displayed a cathepsin B staining pattern spread throughout the cells and outside the boundaries of lysosomes compatible with LMP. In cells treated with SLM plus NAC, we observed a different active cathepsin B pattern, with signal accumulation of cathepsin B in specific places (Figures 6B and S6A). The data are consistent with the hypothesis that SLM treatment triggers an autophagic process that cannot proceed adequately because of LMP resulting from oxidative stress. Addition of NAC, however, enabled, the autophagic flux to continue (Figure 7).

## DISCUSSION

The objective of this work was to elucidate the mechanism of cell death induced by SLM in glioblastoma cells, with the ultimate aim of facilitating rational design of therapeutic intervention for the disease.

There are several hypotheses which could explain the enhanced toxicity of SLM in GSC. One reasonable explanation is that the GSC compartment has a diminished ability to manage cation exchange. Recently, several authors have found an association between Na^+^/K^+^ exchange and stem/progenitor cell characteristics and also poor prognosis, such as, aggressive and resilient tumoral growth [36, 37]. An implication of this hypothesis is that disruption of the balance of Na^+^ and K^+^, as brought about by SLM, could be therapeutic.

Understanding the point where the cell will inexorable die (also called Point of Not Return: (PONR)) could help us to understand why SLM is so efficient at triggering cell death. In our model, the PONR was before the increase in ROS levels and could have been associated with processes in mitochondria. We propose that the PONR in our system was mitochondrial membrane permeabilization, and that, after MOMP, cell death would be irreversibly triggered through the release of mitochondrial intermembrane proteins such as Cyt C or AIF, which would determine the mode of cell death: apoptosis or programmed necrosis. In relation to this line of thinking, note that other authors have proposed that mitochondrial membrane permeabilization be regarded as a checkpoint for apoptosis or cell death by necrosis [38]. In our model, SLM triggered necrosis, and we hypothesize that this is because apoptosis is inhibited by a lack of intracellular ATP resulting from mitochondrial dysfunction. Mitochondrial membrane permeabilization results in programmed cell death, and for this reason we propose that the cell death that occurred in our experimental system is best described by the term *programmed-regulated necrosis*. There are several modalities of programmed-regulated necrosis that include necroptosis [28], which is the best characterized process. Without the evaluation of necrosome formation or death receptors activity it is not possible to conclude that this is the submodality of necrosis that takes place in our system.

That SLM increases ROS levels has been demonstrated by several authors [13, 16, 17, 39], which suggest that high levels of ROS are an overall consequence of the SLM treatment. We propose that oxidative stress is produced by a low mitochondrial ΔΨ. A large leak in electron transport is known to generate superoxide anion [40, 41], and these radicals can rapidly dismutate to H_2_O_2,_ which worsens the redox balance [42]. We demonstrated that although oxidative stress did not interfere with cytotoxicity in our model, it did play an important role in determining the mechanism of cell death. Reducing oxidative stress with NAC reduced the ER stress and the UPR. Studies by other researchers indicate that ROS can induce an increment of unfolded proteins, and when this happens the UPR serves as an anti-oxidant mechanism that can degrade the oxidized unfolded proteins [43]. In parallel to its effect on the UPR, we found that oxidative stress inhibited autophagy. We observed that lowering the oxidative stress decreased the levels of p62 (Figures 5B and S5A), which implies restoration of autophagic flux. Another consideration to be taken into account is that an increase of ROS levels can alter lysosomal lipids, and this could affect lysosome-autophagosome fusion [44, 45]. In our study, in cultures treated with SLM plus NAC there was frequently co-localization of cathepsin B and Lamp1 (Figures 6B and S6B), and so LMP had not occurred in the majority of cells.

On this basis, we propose that in our model autophagic flux in SLM-treated cells was blocked by lipid oxidation due to oxidative stress. Importantly, oxidative stress could be instructing the type of cell death in SLM-treated cells, suggesting that cell death modality is a dynamic concept which depends on the cellular stresses and the cellular mechanism activated.

## MATERIALS AND METHODS

### Cell lines and culture conditions

The adult cell lines GSC23, GSC11, GSC7-2, GSC2-27, GSC5-22, GSC2, GSC231, GSC7-11, GSC10-6, GSC11-28 and GSC229 were kindly provided by Dr. Lang (Department of Neurosurgery; MD Anderson Cancer Center, USA). BTSC lines were maintained as neurospheres [47]. The pediatric cells line SF188, KNS42, RES259, RES186 were kindly provided by Dr. Jones (Institute of Cancer Research, UK). The established cell lines: U87 MG, U373 MG, U251 MG and T98G were obtained from the ATCC. Attached cell cultures were maintained in Dulbecco's modified Eagle/F12 medium (1:1, vol/vol) supplemented with 10% FBS (Fetal Bovine Serum, Thermo Fisher Scientific Inc, Waltham, MA). All the cell lines were grown in a humidified atmosphere of 5% CO_2_ at 37ºC.

### Reagents

Cells were treated with different compounds: SLM (Sigma-Aldrich, St Louis, MO), TMZ (Dpt. of Pharmacy, University Hospital of Navarra), BafA1 (Sigma-Aldrich, St Louis, MO), RAD001 (Everolimus, Selleck Chemicals, Houston, TX), Nife (Sigma-Aldrich, St Louis, MO), NAC (Sigma-Aldrich, St Louis, MO). Each of these reagents was resuspended according with the manufacturer's instructions.

### Cell viability assay

Cell lines were seeded at a density of 5 × 10^3^ cells per well in a 96-well plate. Cells were treated with SLM and/or TMZ at concentrations ranging from 1 × 10^−9^ M to 1 × 10^−3^ M. Seven days later, cells were incubated with MTT (Sigma-Aldrich, St Louis, MO) for 2 h at 37ºC. Afterwards, supernatants were removed and DMSO (Sigma-Aldrich, St Louis, MO) was added (50 μl) to each well. Absorbance was measured at a wavelength of 540 nm in a Sunrise microplate reader with Magellan software (Tecan, Männedorf, Switzerland).

### ATP assay

After the indicated treatments, cells were rinsed three times with PBS and counted in a Neubauer chamber (Celeromics, Grenoble, France) for the subsequent standardization. Boiled deionized water was used to wash away broken cellular membranes and inhibited ATPase molecules [48]. After vortexing and centrifuging the mixture (4ºC, 12,000 g for 5 min), a 20 μL suspension of each sample was quantified by bioluminescence with the ATP kit from Molecular Probes (Invitrogen). The reagents and reaction mixture were combined according to the protocol of Molecular Probes; Bioluminescence was detected using Lumat LB 9507 (Midland, Canada).

### Determination of mitochondrial membrane potiential, ΔΨ, using the Rhodamine-123 probe

Cells that had previously been treated with the reagents tested were incubated with Rhodamine-123 (RH-123) (10 μM) during 30 min at room temperature with protection from light. Cells were then rinsed twice with medium and incubated for an additional 30 min with medium supplemented with serum. After this time 10^6^ cells/ml were resuspended in PBS (500 μl) and PI 5 μM. Membrane potential was measured with a FACSCalibur (Becton Dickinson, San Jose, CA) using the CellQuest software.

### Measurement of ROS production using the 2′,7′-dichlorofluorescein diacetate probe

Cells were plated in 6-well plates and incubated with the different treatments. 48 hours later, cells were collected by centrifugation (5 min, 1500 rpm) and resuspended in whole medium. Then cells were incubated with the probe −2′, 7′-dichlorofluorescein diacetate (DCF-DA; from Molecular Probes (Invitrogen) (40 μg/ml) a 37ºC. At this point cells were rinsed twice with PBS and then analyzed by flow cytometry.

### Immunoblotting analysis

After the indicated treatments total cell proteins were extracted on ice with buffer lysis (1% tween in PBS) in the presence of freshly added protease and phosphatase inhibitors. Protein concentration was determined using the Bradford method. A total of 40 μg/lane protein extract was separated by Tris/Glycine SDS-PAGE and transferred to nitrocellulose membrane (Bio-Rad Laboratories; Hercules; CA). Non-specific binding was blocked with non-fat milk 5% in PBS-T for 1 h at room temperature. Blots were incubated with the following antibodies: P62 (Sigma-Aldrich, St Louis, MO), LC3A/B, BIP, ATF4, P-elF2α, CHOP, caspase 3, cleaved caspase 7, P-H2A.X (Cell Signaling Technology, Danvers, MA), cathepsin B (Merck, Darmstadt, Germany), and Lamp1 (Abcam plc, Cambridge, UK). As housekeeping markers we used GAPDH (Abcam plc, Cambridge, UK), GRB2 (BD Transduction Laboratories^™^, San Jose, CA) and α-Tubulin (Sigma-Aldrich, St Louis, MO). Amersham's enhanced chemiluminescence protocol (Perkin Elmer Waltham, MA) was used to develop membranes.

### Caspase assay

After the indicated treatments, cells were incubated with Caspase-Glo Reagent and, after one hour, bioluminescent fluorescence was detected using spectrofluorometer (SpectraMAX gemini XS, Molecular devices). Luminescence is proportional to the amount of caspase activity present, and so the proportional fluorescence intensity of treated cells relative to non-treated cells enables determination of differences in calpain activity.

### Quantitative real-time PCR experiments

The relative levels of RNA messenger of: p62, Musashi, Sox2 and Nestin were standardized with 18S ribosomal RNA (18S), by real time PCR (RT-PCR) using an ABI 7700 sequence detection system (Applied Biosystems, Foster City, CA). The expression levels relative to 18S were calculated using the ddCt method [49], *P* values were calculated by analysis of variance using Microsoft Excel. Primer sequences used were as follows: P62 (forward primer 5′- GCACCCCAATGTGATCTGC -3′; reverse primer 5′-CGCTACACAAGTCGTAGTCTGG -3′), Mushashi (forward primer 5′-GGGACTCAGTTGGCAGACTAC-3′; reverse primer 5′-CTGGTCCATGAAAGTGACGAA-3′), Nestin (forward primer 5′-CTGCTACCCTTGAGAC ACCTG-3′; reverse primer 5′-GGGCTCTGATCTCTGC ATCTAC-3′), Sox2 (forward primer 5′-ACCGGCGGCA ACCAGAAGAACAG-3′; reverse primer 5′-GCGCCG CGGCCGGTATTTAT-3′).

### Immunofluorescence analysis

Glioblastoma cells were cultured on glass coverslips and fixed with methanol. Samples were blocked in phosphate-buffered saline/fetal bovine serum 10%. Cells were then incubated with antibodies directed against the following proteins: AIF (Abcam plc, Cambridge, UK), Lamp1 and Cathepsin B (Merck KGaA, Darmstadt, Germany) for 1 h at room temperature. Afterwards, samples were incubated with secondary antibodies: Alexa Fluor 594, monkey anti-rabbit and/or Alexa Fluor 488 donkey anti-mouse (Thermo Fisher Scientific Inc, Waltham, MA). All antibodies were used in accordance with the manufacturers' instructions. The cover slips were mounted with mounting medium with DAPI (Vector Laboratories INC, Burlingame, CA). Finally, the fluorescence signals were visualized and digital images were obtained with the fluorescence microscope Zeiss Axioplan 2ie (Zeiss International, Germany).

### Statistical analysis

For MTT assay analysis, the IC_50_ value (the dose that causes 50% of affected cells to die, i.e., the dose that resulted in 50% cell survival) was calculated with the CalcuSyn Software (Biosoft, Cambridge, UK). Experiments were performed three times with each reagent treatment administered in quintuplicate. All data are expressed as ± standard deviation. Comparisons were made with two-tailed parametric and nonparametric tests (Student's *t*-test and Mann-Whitney U test, respectively). The statistical program used was GraphPad Prism (Graphpad Software, San Diego, CA).

## SUPPLEMENTARY MATERIALS FIGURES


