# Supplementary material for: Salinomycin induced ROS results in abortive autophagy and leads to regulated necrosis in glioblastoma
Source: Oncotarget. 2016 Apr 21;7(21):30626–41. doi: 10.18632/oncotarget.8905 (PMC5058706; doi:10.18632/oncotarget.8905)
Supplement: Supplementary file 1 [file oncotarget-07-30626-s001.pdf]

Salinomycin induced ROS results in abortive autophagy and leads to regulated necrosis in glioblastoma

Supplementary Materials

MATERIALS AND METHODS

Self-renewal analysis

Cells derived from the dissociation of clonal single neurospheres were seeded in 96-well plates and the number of secondary spheres generated was assessed after 8–10 days. In order to avoid including the colonies that may have been formed by transiently amplifying cells in these cultures, only secondary spheres that exceeded 120 µm in diameter were counted. To confirm that the latter were, indeed, formed by stem cells, at least 15 individual secondary spheres were randomly selected and subjected to further, long-term (2 months) propagation in each subcloning experiment.

Neurosphere size analysis

For quantification of neurosphere size, cells derived from the dissociation of clonal single neurospheres were seeded in 96-well plates, and the size of the generated secondary spheres was assessed after 10 days. We counted 20 neurospheres per sample, and the neurospheres size media was plotted with 95% confidence intervals (CIs). Images were captured and measured using a deconvolution microscope (Zeiss, Germany).

Flow cytometric analysis of apoptosis

The membrane and nuclear events during apoptosis were analyzed by flow cytometry using FITC-Annexin V and propidium iodide (PI) staining. Following treatment, SF188 or GSC11 cells were harvested and centrifuged for 10 min at room temperature at 3000 rpm. Cells were washed with PBS and resuspended in binding buffer, and then 5 µl Annexin V-FITC (20 µg/ml) and 5 µl PI (50 µg/ml) were added. After incubating in the dark for 15 min, the samples were analyzed by flow cytometry (Becton-Dickinson). The assay was performed with a two-color analysis of FITC-labeled Annexin V binding and the uptake of PI. Living cells (Annexin V−/PI−, Q3), early apoptotic cells (Annexin V+/PI−, Q4), late apoptotic cells (Annexin V+/PI+, Q2), and necrotic cells (Annexin V−/PI+, Q1) were distinguished. Therefore, the total apoptotic proportion included the percentage of cells with fluorescence for Annexin V+/PI− and Annexin V+/PI+.

Quantification of acidic vesicular organelles (AVOs)

After treatment with the tested reagents, cells were stained with acridine orange (1 µg/ml for 15 min) (Sigma-Aldrich). After three rinses with PBS, cells were analyzed by flow cytometry. Green (510–530 nm) and red (> 650 nm) fluorescence emission from 10^4 cells illuminated with blue (488 nm) excitation light was measured with a FACSCalibur (Becton Dickinson, San Jose, CA) using the Cell Quest software.

Transmission electron microscopy

The ultrastructural analysis of cell morphology after the indicated treatments was performed using transmission electron microscopy (TEM). Treated cells were centrifuged (5 minutes at 3000 rpm) and rinsed with PBS. Samples were fixed with a solution containing 3% glutaraldehyde and 2% paraformaldehyde in 0.1 M cacodylate buffer, pH 7.3. Samples were then washed and treated with 0.1% Millipore-filtered, cacodylate-buffered tannic acid, postfixed with 1% buffered osmium tetroxide for 30 min and stained in block with 1% Millipore-filtered uranyl acetate. The samples were dehydrated with increasing concentrations of ethanol, infiltrated, and embedded in LX-112 medium. After this, they were polymerized in a 60º C oven for 2 days. Ultrathin sections (65 nm) were cut in a Leica Ultracut microtome, stained with uranyl acetate and lead citrate in a Leica EM Stainer, and examined in a Jeol 1210 transmission electron microscope (Jeol Ltd., Herts, UK).

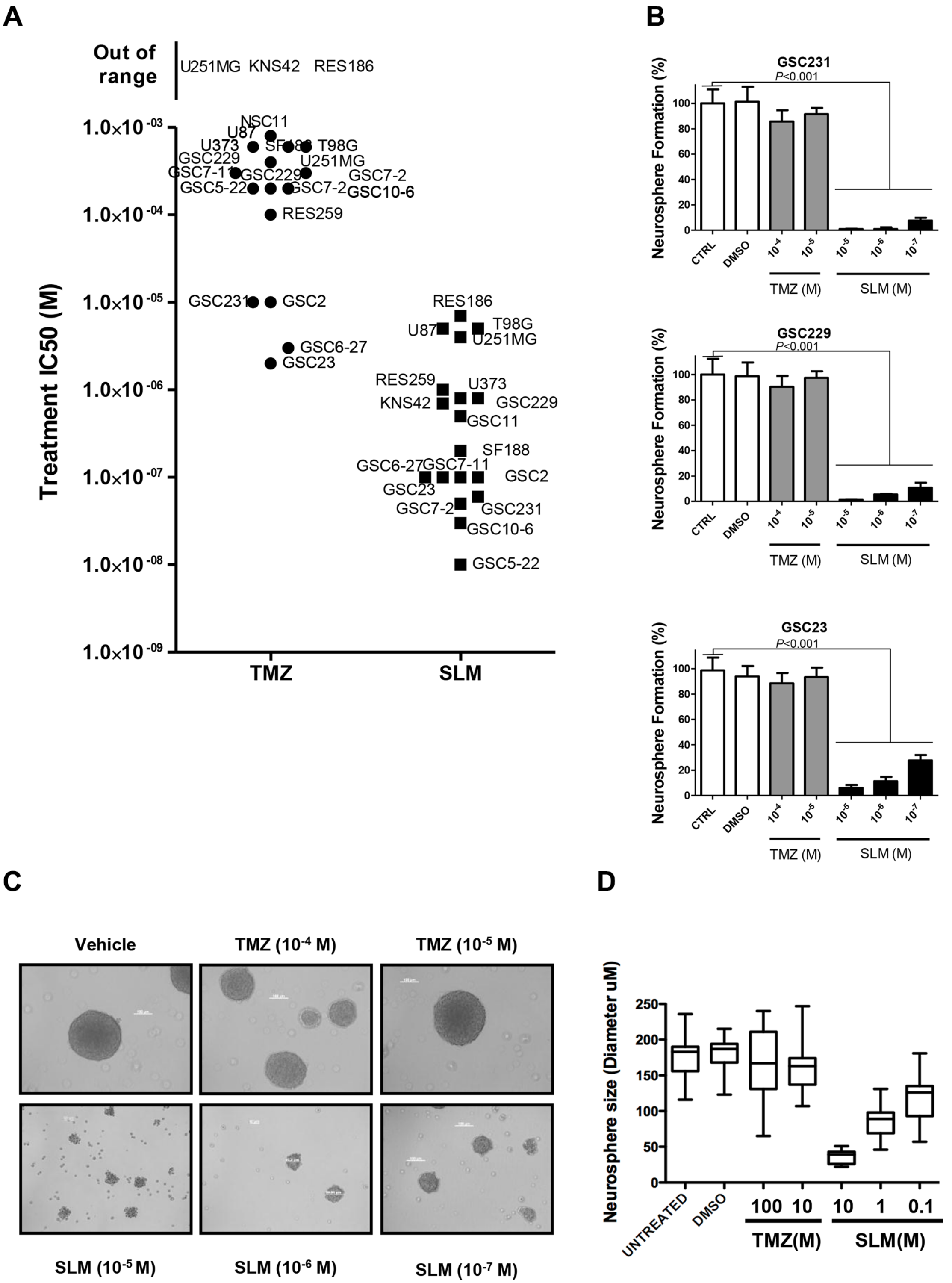

Supplementary Figure S1: **(A)** Median-effect doses (IC50) of SLM and TMZ in attached cell lines and neurosphere cultures. IC50 is the median-effect dose (the dose causing 50% of cells to be affected; here, this is equivalent to 50% survival). The results are expressed as mean values from Figure 1A. **(B)** GSC231, GSC229 and GSC23 were treated with TMZ or SLM at the indicated concentrations and the number of secondary spheres generated was assessed after 10 days and expressed as percentages of the numbers of spheres in non-treated cells. To confirm that the spheroids were formed by stem cells, we randomly selected at least 15 individual secondary spheres and subjected them to further, long-term (two-month) propagation. **(C)** Representative micrographs illustrating neurosphere size (the scale is 50-µm). Neurosphere size evaluation in GSC11 cells treated with TMZ or SLM at the indicated concentration (µM). **(D)** Neurosphere size evaluation in GSC11 cells treated with TMZ or SLM at the indicated concentration (µM).

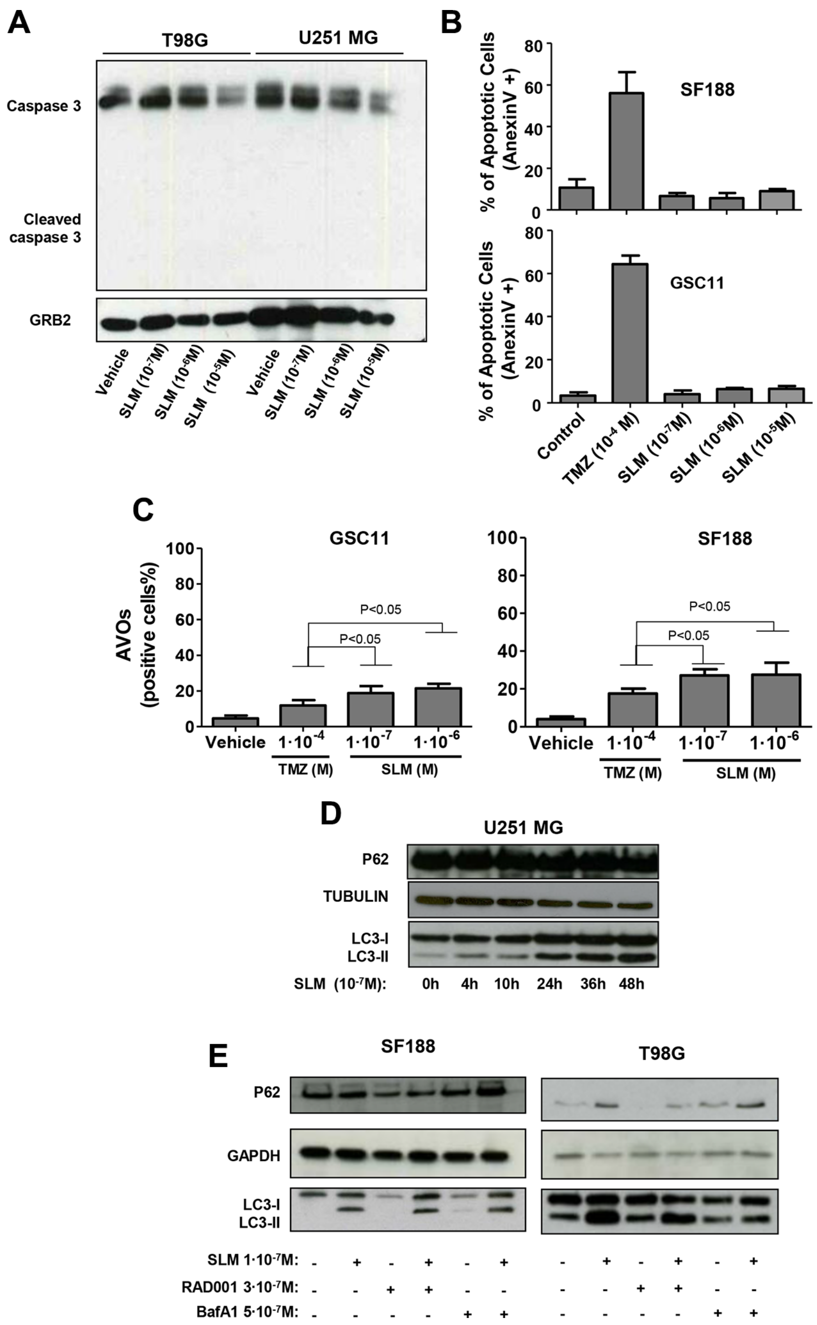

Supplementary Figure S2: **(A)** T98G and U251MG cells were seeded at a density of 1×10^5 cells per well in 6-well plates. After 24 h of culture, cells were incubated with SLM at the indicated concentrations. Cells were collected 48 hours later and subjected to western blot analyses. Shown is a representative western blot of three independent experiments. **(B)** Analysis of early apoptotic cells after treatment with either SLM or TMZ at the indicated dosages. GSC11 or SF188 cells were stained with 5 µl Annexin V-FITC (20 µg/ml) and 5 µl PI (50 µg/ml) were added and subjected to flow cytometric analysis. The results are expressed as mean percetange values ± SD from three independent experiments. **(C)** Analysis of acidic vesicular organelles (AVOs) after treatment with either SLM or TMZ at the indicated dosages. GSC11 or SF188 cells were stained with acridine orange (1 µg/ml) and subjected to flow cytometric analysis. The results are expressed as mean values ± SD from three independent experiments. **(D)** U251 cells were seeded at a density of 1×10^5 cells per well in a 6-well plate. The following day cells were incubated with SLM. Cells were collected at the indicated times. Levels of protein expression were analyzed by western blot using antibodies against LC3-I/II and P62. α-tubulin was used as loading control. The western blot shown is representative of three independent experiments. **(E)** SF188 and GSC11 cells were seeded at a density of 1×10^5 cells per well in a 6-well plate. The next day, cells were incubated with SLM and/or TMZ at the indicated concentrations. Cells were collected 48 hours later, and the levels of protein expression were analyzed by western blot using antibodies against LC3-I/II and P62. GAPDH was used as loading control. The western blot shown is representative of three independent experiments.

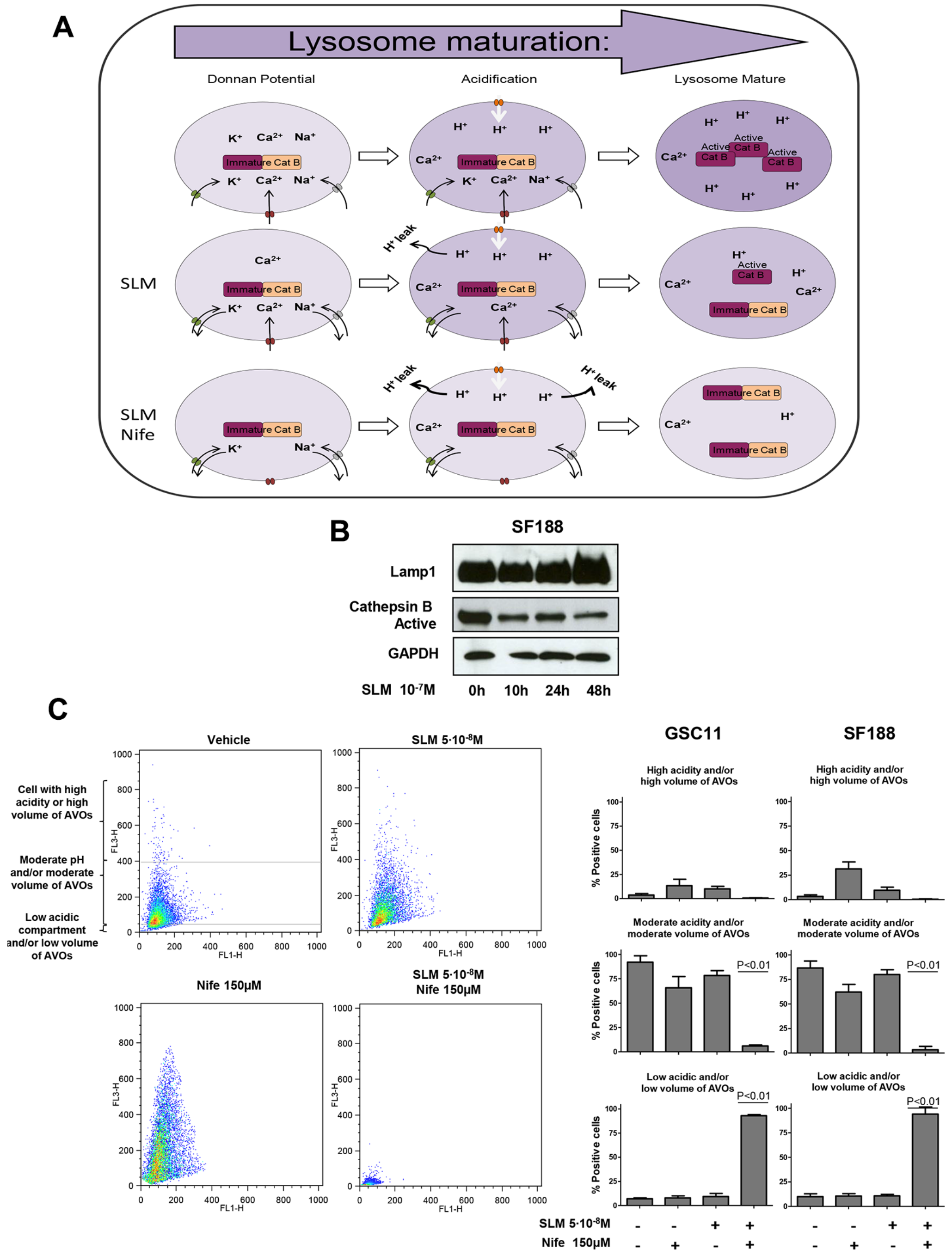

Supplementary Figure S3: (A) Scheme depicting the process of lysosome maturation after SLM or SLM plus Nife treatment. Lysosome maturation requires the Donnan potential in order to retain inside lysosomes the protons needed for acidification. Lysosome acidification provides the optimum environment for maturation of cathepsins. Cells treated with SLM did not retain the cations K+ and Na+ in the lysosome. If the Donnan potential drops, protons cannot be kept inside the lysosomes, and, as a consequence, acidification cannot be adequately completed and the maturation of cathepsin B is inefficient. In cells treated with SLM plus Nife, the Donnan potential was practically absent; there would be proton leak from the lysosomes, insufficient acidification, and disruption of cathepsin B maturation. **(B)** SF188 cells were incubated with SLM (1×10^{-7} M) and collected at the different times stated. Samples were analyzed by western blot for Lamp-1 and cathepsin B. GAPDH was used as the loading control. The western blot shown is representative of three independent experiments. **(C)** GSC11 and SF188 were seeded at a density of 1×10^5 cells per well in 6-well plates. After 24 hours of culture, cells were incubated with SLM and/or Nifedipine (Nife) at the indicated concentrations. Cells were collected 48 hours later, and the content of acidic vesicles (AVOs) was analyzed. Samples were stained with acridine orange (1 µg/ml) and subjected to flow cytometry analysis. Left-hand side: data obtained by flow cytometer. Right-hand side: graphical representation of results (expressed as mean values ± SD) obtained from three independent experiments.

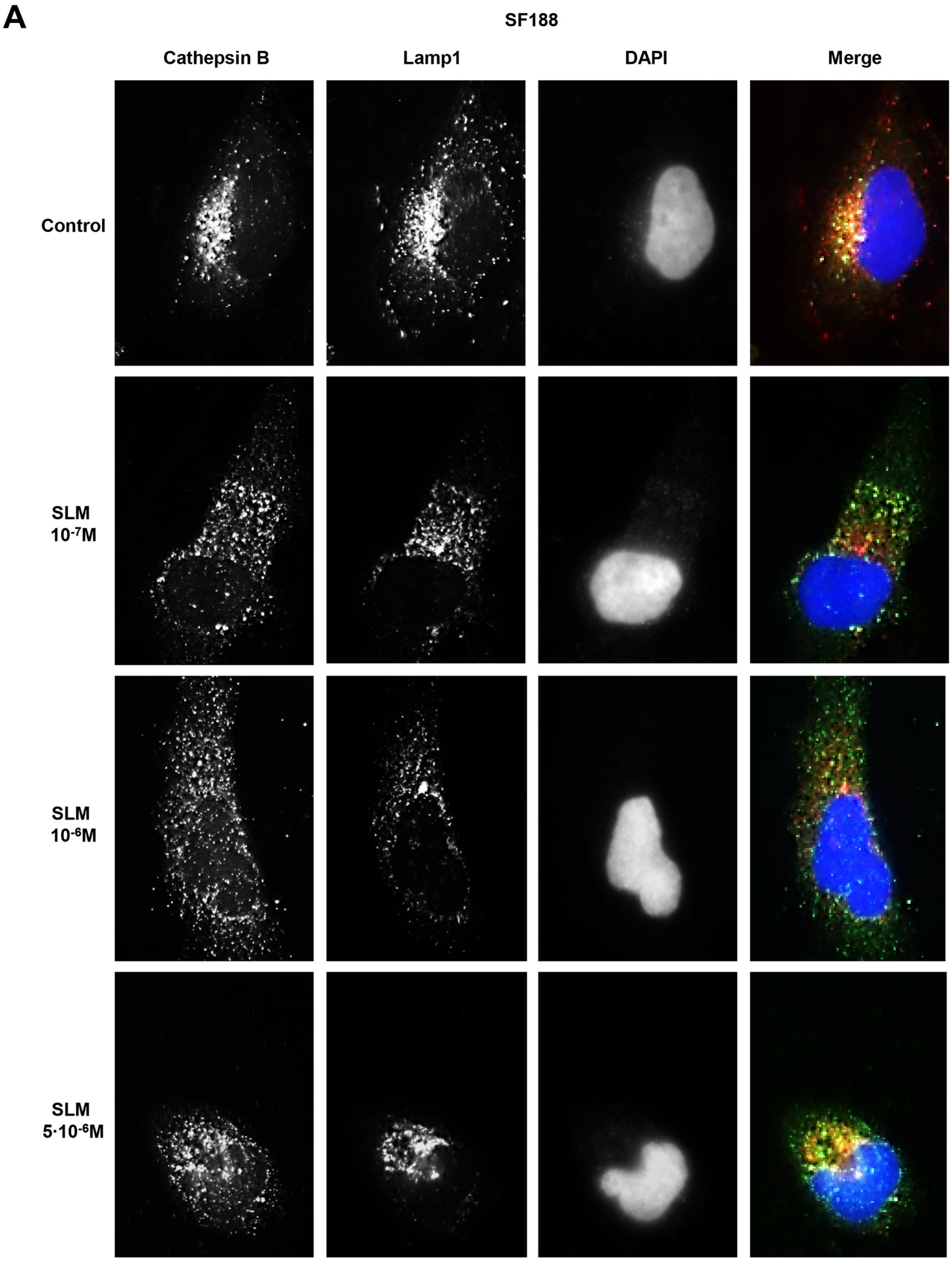

Supplementary Figure S4: (A) SF188 cells were treated with SLM at the indicated concentrations. Samples were collected and fixed after 48 of treatment. Cathepsin B (green) and Lamp1 (red) localization was evaluated by immunofluorescence. Representative images of three independent experiments are shown. DAPI (blue) was used for nuclear staining. The micrographs shown are representative of the morphology of treated cells (×690 magnification).

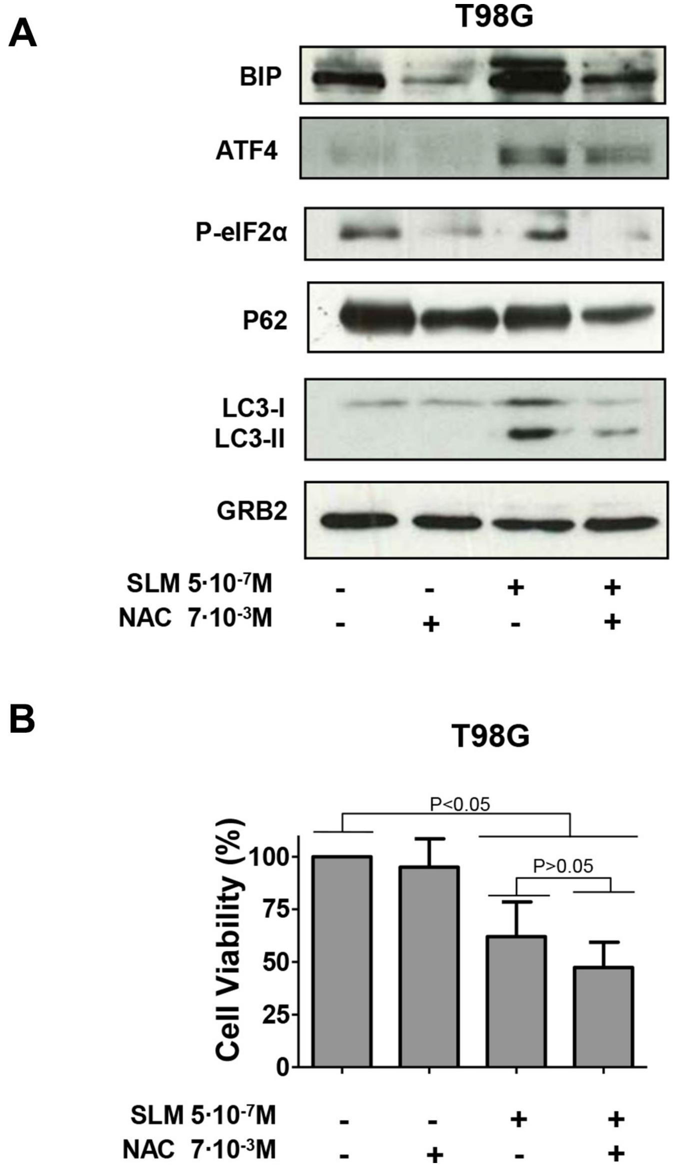

Supplementary Figure S5: **(A)** GSC11, SF188, and T98G cells were seeded at a density of 1×10^5 cells per well in a 6-well plate. The following day, cells were incubated with SLM, NAC or both drugs at indicated doses. Cells were harvested three days later, and protein samples were analyzed by western blot for BIP, ATF4, P-elF2α, P62 and LC3-II conversion. GRB2 was used as loading control. The western blot shown is representative of three independent experiments. **(B)** GSC11, SF188, and T98G cells were seeded at a density of 1×10^5 cells per well in a 6-well plate. The following day, cells were incubated with SLM, NAC or both drugs at indicated doses. Three days later cells were counted in a Neubauer chamber. Results are expressed as percentage of cell viability relative to non-treated cells. The mean ± SD from three independent experiments is shown.

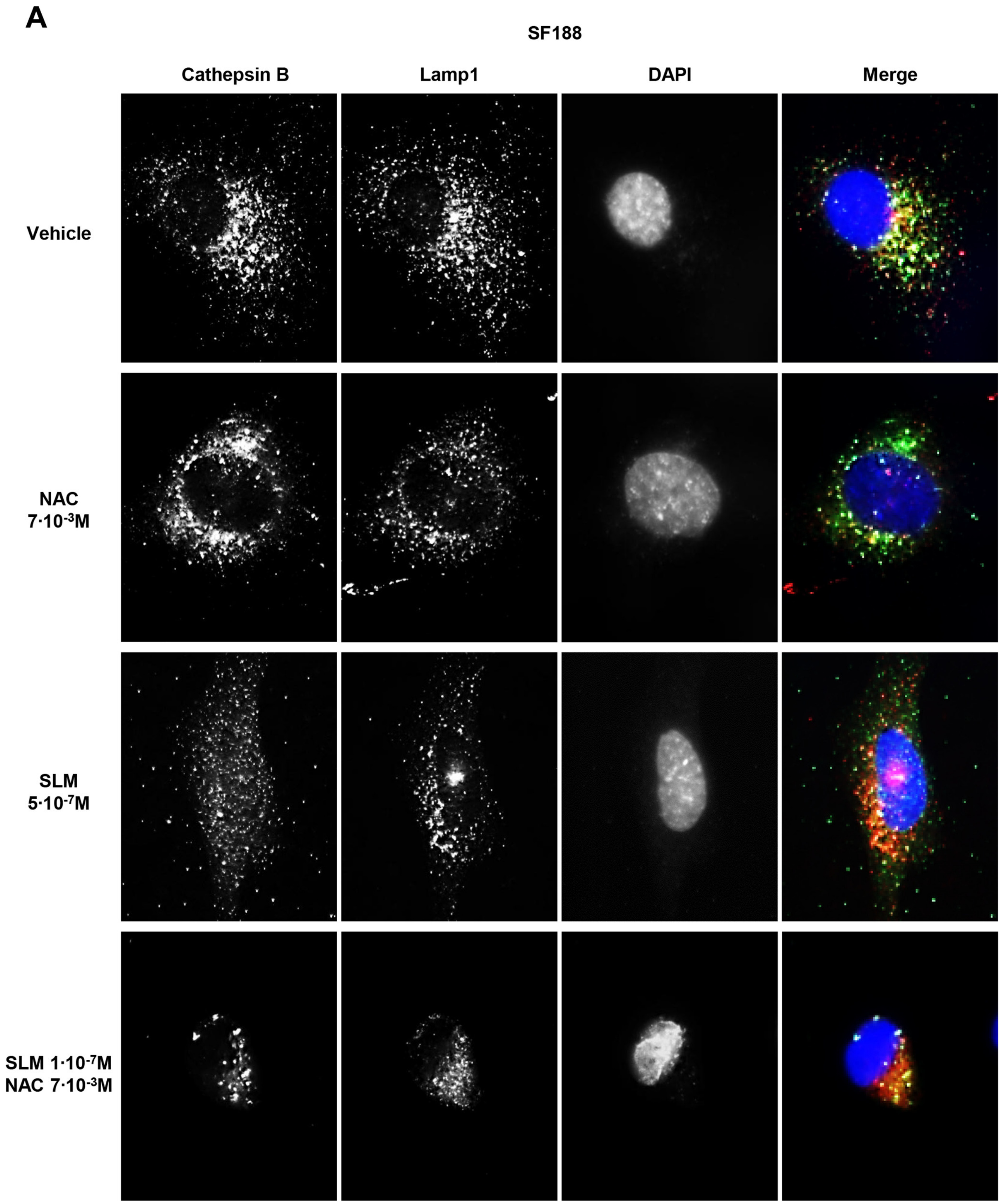

Supplementary Figure S6: (A) SF188 cells were incubated with SLM, NAC, or both at the indicated concentrations. 48 hours after drug administration, cathepsin B (green) and Lamp1 (red) localization was determined with immunofluorescence. Representative immunofluorescence images for three independent experiments are shown. DAPI (in blue) was used for nuclear staining.
